# Continuous learning in multiple sclerosis care: a qualitative study of the expanded learning model for systems

**DOI:** 10.5116/ijme.5cfa.29cb

**Published:** 2019-06-28

**Authors:** Dana Ravyn, Beth Goodwin, Rob Lowney

**Affiliations:** 1 CMEology, West Hartford, Connecticut, USA

**Keywords:** Multiple sclerosis, continuing education, learning theory, qualitative, coordination of care, systems, healthcare environment

## Abstract

**Objectives:**

This study characterized how an online continuing education activity affected knowledge, attitudes, and practices of healthcare professionals who care for patients with multiple sclerosis (MS) and whether those changes reflected theorized translational mechanisms proposed in The Expanded Learning Model for Systems (TELMS).

**Methods:**

This preliminary study used semi-structured interviews (thematic analysis) to assess whether and how translational mechanisms underpinning the TELMS theory might be revealed in learners’ attitudes and practice behavior. Eighteen participants (primarily neurologists and nurses) were interviewed by telephone or online. Thematic analysis identified relevant themes according to sensitizing concepts derived from TELMS and the recognition of emergent themes.

**Results:**

Textual interpretation of interview data revealed that MS providers act in various scenarios that validate the principles of TELMS model of learning engagement. Further, elements of translational mechanisms proposed by TELMS were consistently observed in the narrative reflections. Emergent themes included the importance of practices such as goal setting, coordination of care, systems-level MS care, and economic considerations. Practitioners particularly drew on ideas from TELMS when facing challenges in diverse cultural and sociocultural settings.

**Conclusions:**

We identified mechanisms of change reflected in the TELMS model that is useful for the design and evaluation of future educational activities. These include attitudes and beliefs about the application of evidence-aligned MS care, as well as the commitment to multidisciplinary strategies, enhanced coordination of care, and promotion of systems-based changes. Future studies are needed to further validate the TELMS model.

## Introduction

Healthcare delivery operates within elaborate environmental contexts that influence the behavior of patients and healthcare providers.^1^^-^^3^ Patients and providers who operationalize health decisions will be influenced by the unique sociocultural nature and institutional constraints of the setting in which their decisions are made.^4^ The dynamics underlying these phenomena have long been of interest to those seeking to improve the efficiency of healthcare delivery.^5^ Continuing education (CE) is a phenomenon largely enveloped by the healthcare delivery environment while at the same time having unique characteristics and influences.  Further, current models of education for healthcare professionals often focus on individual behavior. As a result, researchers and educators have been interested in whether CE can not only affect individual behavior, but also have an impact on teams, organizations, and systems. In 2015, a seminal whitepaper was published that provided a new model for learning in healthcare systems.^6^ In the Expanded Learning Model for Systems (TELMS) for continuing education, Ruggiero and colleagues stressed that systems characteristics such as structural contexts, workplace culture, and organizational processes are important considerations in the design and implementation of educational initiatives that strive to transform practice in context.^6^ Understanding complex behavioral factors that influence utilization of healthcare can lead to enhancements in health quality, such as those aligned with the “Triple Aim,” which include improving the experience of care, improving the health of populations, and reducing costs.^7^ Further, the healthcare environment is changing, with more complex and prevalent chronic diseases requiring greater interaction between primary and specialist providers.^8^ As a result, new delivery models are needed, such as the patient-centered medical home and care coordination. These evolving paradigms emphasize the role of patient and provider education and the central role of a team-based approach.^9^ Models are needed to elucidate the role of CE as an integral force in the change of processes in healthcare.^10^  TELMS posits that insights from behavioral and social science research suggest a series of translational mechanisms that inform continuous learning in a complex system. As a result, TELMS emphasizes the benefit of systems-based change that is integrated across healthcare delivery structures.

While TELMS advances the theoretical approach to implementing and evaluating programs designed for systems-based interventions, systems-level programs are complex and costly.  In practice, most CE takes place in an individual-level learning environment. Studies are needed to elucidate the role translational mechanisms of TELMS play in learning to better appreciate the validity of TELMS and its significance in the design and evaluation of diverse CE interventions. It is particularly important to examine how the translational mechanisms of TELMS might contribute to attitudes, beliefs, and behaviors that lead to systems-level practice change when the education is limited to an individual level intervention. It was the objective of this study to address this gap. We evaluated an online learning activity to improve care for patients with multiple sclerosis (MS) that applied selected principles of TELMS in its design and implementation. In this preliminary study, we identified how translational mechanisms behind the TELMS theory influenced learners’ attitudes and behavior toward systems-level needs and changes in the care of patients with MS.

## Methods

### Educational intervention

The online CE learning activity, Treatment Decision Making, and Achieving Therapeutic Goals in Multiple Sclerosis: Individual and Systems-Level Advances, was released in September 2017 and was available online for 12 months. The activity was certified for physicians and nurses by Postgraduate Institute for Medicine.

The activity consisted of three 30-minute modules on the following topics: (1) pathophysiology of MS, (2) evaluation and management of suboptimal treatment response, and (3) identifying treatment goals and personalizing therapy. The target audience was neurologists, neurology advanced practice clinicians, and other healthcare providers who manage patients with MS. The educational design and content were formulated with consideration of TELMS principles. Specifically, the activity endeavored to improve awareness of deficits or gaps in evidence-based care delivery, enhance personal commitment among teams, encourage conversion of information into practice behaviors, engage patients and caregivers and other healthcare providers, promote and reinforce coordination of care, and emphasize the importance of cost-effective care. Several methods were employed to evaluate the outcomes of the activity. This summary focuses on qualitative semi-structured interview results.

### Study design

The overarching goal of this study was to characterize how a CE activity affected knowledge, attitudes, and practices of healthcare professionals involved in the care of patients with MS and whether those changes reflected theorized translational mechanisms proposed in TELMS.^6^ The translational mechanisms of the TELMS model are provided in Table 1. The study design included a qualitative analysis of semi-structured interviews. The objectives of the qualitative study were to characterize the relationship between an existing conceptual framework and the attitudes and practices of providers, as well as to deepen understanding about the subject by identifying emerging themes. We employed hybrid inductive and deductive qualitative analysis guided by a conceptual framework.^11^^,^^12^ This directed approach uses sensitizing concepts from extant research to create the initial coding schemes or themes and strives “to validate or extend conceptually a theoretical framework.”^13^

**Table 1:** The Expanded Learning Model for Systems; summary of the key stages and mechanisms of the model(6)

Behavior and Learning Stages	Patient, Caregiver, and Healthcare Professional Aims	Translational Mechanisms
Activate	Improve awareness	When a problem is identified, individuals desire to improve their awareness of the issues relevant to them.
Advance	Convert information	Through analysis of their situation, individuals aim to convert information into practical actions that address their needs.
Aspire	Demonstrate engagement	Individuals pursue some form of useful engagement to practically address problems or reinforce beneficial actions.
Allocate	Substantiate partnerships	To maintain beneficial engagement, individuals develop effective partnerships and communities.

Participants were identified by volunteering for an interview when completing the online evaluation at the completion of the activity. A semi-structured interview was conducted by telephone following informed consent. In some cases, participants completed a written online survey based on the same open-ended questions used in the interview. Survey questions were administered to the point where there were redundancy and lack of new themes in the course of interviews. Survey respondents received a gift card for their participation.

### Data analysis

Audio recordings were transcribed verbatim. Deidentified transcripts or online survey responses were read and coded by two investigators (DR and BG). Interviews and notes were coded using Dedoose software.^14^ Thematic analysis was initially performed by DR, then reviewed and revised by all investigators.

### Human participants

The nature and risks/benefits of participation were explained to individuals participating in the interview and all participants provided consent prior to interviews. All participant data were deidentified. The study was reviewed by an independent institutional board (Solutions IRB) and the research was verified to be an exempt educational survey according to US 45 CFR 46.101(b)(2).

## Results

A total of 5854 healthcare professionals participated in the learning series. The participants included primarily physician neurologists (n=2864), nurses (n=1093), and other professionals involved in the care of patients with MS (n=1879) such as neurology non-physicians and physicians in specialties other than neurology. Most participants (76%) had been in practice for more than ten years. The majority of participants reported neurology or MS specialist as their specialty and the most common practice settings were metropolitan hospitals (71%); most worked in group practices (84%). Of the 18 participants who completed the interview, 11 were interviewed by telephone, and 7 completed the written interview online. Interview participants included six physicians, five nurse practitioners, six registered nurses, and one physician assistant.

Sensitizing concepts that guided the initial coding were corroborated by qualitative analysis of semi-structured interview data. Emergent concepts supported the principal themes in the qualitative observations. For example, the thematic analysis revealed the importance of practices such as team goal setting, coordination of care, MS care on the systems level, and economic considerations. The following excerpts demonstrate representative findings supporting the major themes of TELMS (Table 1) identified from the qualitative results.

### Activate

Responses from interviews were analyzed to determine whether learners were more informed about problems leading to professional practice gaps and whether education activated learners to be more informed and knowledgeable about evidence-based shortcomings in MS care within their healthcare system.^6^

Participation in the activity increased awareness of issues such as inadequately addressing therapy tolerability, not starting disease-modifying therapies (DMTs) soon enough, and adherence challenges of patients. Several providers emphasized a lack of education on the part of providers and patients regarding treatment options for relapsing MS, as well as in overall education about MS and understanding of treatment options.

“I just don't believe there is a good understanding of all the options with treating relapsing adults.” (RN, internal medicine)

“…I believe that it’s driven by basically patients’ lack of knowledge.” (NP, neurology)

The complexity of treatment options appeared to be a vexing problem in terms of both educating patients and facilitating provider decision making, such that clinicians struggle to help patients choose the right therapy.

“…We have over a dozen disease-modifying therapies, and each one of them obviously has an impact point on the immune system, but why do we need so many of them?” (MD, neurologist)

Nevertheless, learners suggested an increased personal commitment to overcoming some of these gaps.

“I have taken it upon myself to learn much more about it, become much more in-depth with the education…because it is such an in-depth disease process, and there are so many aspects to it.” (RN, neurology)

Considering their awareness of limitations or gaps in care, providers seemed activated to translate their learning into best evidence-based practices.

“Taking into account the pathophysiological mechanisms and the therapeutic action of new drugs allows us to generate more precise therapeutic action, especially in patients with progressive forms of the disease.” (MD, neurologist)

“If we have a little more on the … information from the MS expert panel that would be very helpful to explain the reasons to start a new medication or not start a new medication.” (PA, neurology)

“I followed each patient's adherence to treatment very closely and changed or modified treatment as needed at each visit.” (MD, neurologist)

“So I certainly think it does help because…it just reminds you of the fact that this is very much an autoimmune or very complex immune process, and an awareness and understanding to keep an open mind about what may be occurring in that new information and data is coming in on MS, and to really think about the patient as a whole, not just as an MS patient.” (MD, neurologist)

### Advance

Learners demonstrated their personal commitment to the second aim of TELMS, converting information into practical actions, which was demonstrated by expressions of personal commitment to greater collegial interaction and team-based care. Informants reported plans for extending learning beyond this activity (such as attending national conferences) and providing the highest level of care. Further, participants felt more informed.

“We [colleagues] have mutual respect and they look to me for knowledge, and that’s why I try to stay on top of things.” (NP, neurology)

“It really helped break it down, and kind of opened my eyes as to how in-depth and broad this is, and I have since done a lot more education on my own and research on my own as to educate myself so that I can help others, fellow colleagues as well as the patients, truly understand what their diagnosis is.” (NP, neurology)

Participants who felt more informed suggested that they had a desire to be aware of their own limitations and described how they might behave differently.

“It definitely helped me feel a little bit more confident in communicating with specialists about MS.” (NP, neurology)

“I know for myself how important it is to differentiate exactly what type of MS they have, and then, to discover with them, do they realize what they have and do they realize the difference in the treatments.” (NP, neurology)

One mechanism by which individuals take practical action is to focus on healthcare systems and systems-based factors that influence MS care.^15^

### Aspire

Participants in this activity found that learning reinforced the value of communication among healthcare providers and between providers and patients. For example, participants emphasized that communication across the multidisciplinary MS team is crucial for the continuity of care. The chief way this is accomplished is by documentation.

Participants identified behavior changes that facilitate engagement and maintenance in the care plan. This was achieved in the context of engagement with patients and other healthcare providers.

“…Even though I’m not the specialist, patients tend to ask still their primary care providers questions about what they should expect whenever they meet the specialist…that gives me a better idea of how to answer this question.” (NP, neurology)

“I felt like I would pay more attention to a patient after the activity. Maybe I would just have a better understanding of the problem they have…” (RN, neurology)

“It will change the way I interact with my patients and with the referring physicians to our clinic because if the medications we see aren’t helping the patient, maybe there is something better out there and they need to be considering something else.” (NP, neurology)

The learning activity encouraged the pursuit of useful engagement by reinforcing actions demonstrated to be or perceived to be beneficial to participants and their patients.

“I am developing a form to be used with our MS patients to document and track treatment goals.” (RN, neurology)

“I thought it would remind the physician to be a little more comprehensive in their approach to the patient.” (MD, neurology)

Once care delivery problems are identified, one way to translate this knowledge into beneficial action is to enhance the coordination of care.^16^^,^^17^ Participants reported increased confidence in the implementation of care-coordination strategies after activity completion. Interview participants were highly attuned to the issue of care coordination. Several informants expressed the difficulties and barriers encountered while trying to coordinate care.

“There was nobody…that I could call to say…can you help get this going on this patient? If we were to have these other resources in place, the outcomes would be much better for these patients.” (NP, neurology)

“Coordination of care…is so much more than just the doctor’s office and the pill that you are taking.  There is homecare; there are different tests, there are special groups and community resources out there, the primary care doctor has to be involved, there is so much more to it than what my knowledge was.” (RN, neurology)

The activity encouraged behaviors that led to better care coordination for both specialists and general practice providers.

“I involved other disciplines as well in managing MS patients; that improved their adherence to the treatment.” (MD, neurology)

“…The activity helped me as far as being more knowledgeable about how to coordinate care…and it also saves them money.” (NP, neurology)

“I think that the main point that I’ve pulled back from this was the coordination of care…it was something that we were definitely lacking, and it’s something that we have been able to pull in slowly since this activity.” (RN, neurology)

### Allocate

On completion of the activity, learners were committed to implementing systems-based care to reduce MS relapses and enhance the cost-effectiveness of care. Healthcare providers demonstrated an interest in substantiating partnerships, such as those exemplified by multidisciplinary care.

“Makes me want to use a lot more of case management.” (MD, neurology)

“…[H]ave always thought and tried to manage patients in a multidisciplinary way; for me, it is clear that this leads to better diagnosis, treatment, and prognosis.” (MD, neurology)

Healthcare providers demonstrated an interest in sharing knowledge.

“I am coordinating with other MS professionals in our area to offer in-office educational seminars.” (RN, neurology)

Concerns about adherence to the care plan and healthcare utilization were common. Participants demonstrated engagement with cost considerations in the way they delivered care.

“It’s hard because we want to do what’s right for the patient, but at the same time, we want to control cost.” (MD, neurology)

The learning activity included an interactive application that allowed users to obtain a visual estimate of the predicted economic impact of preventing relapses in patients with MS. Interview respondents expressed interest in reducing costs of MS care through improved care, earlier interventions, and prevention of relapse.

“So if we can catch things very, very early on…of course, that is going to impact controlling costs.” (NP, neurology)

“Healthcare providers play an important role in …to prevent exacerbations in a disease state, thus minimizing hospitalizations and other healthcare costs.” (NP, neurology)

“I think certainly we are at the forefront of controlling costs.” (MD, neurology)

“I think we have been able to adopt or start some of those systems that render… better care of the patient perhaps at a reduced cost.” (MD, neurology)

## Discussion

This qualitative analysis evaluated data from healthcare providers involved in managing patients with MS to characterize better translational mechanisms that influence behavior and learning stages according to the TELMS paradigm.^6^ TELMS was developed to describe learning engagement, CE implementation, and evaluation at the level of teams, organizations, and systems.^6^ Little is known about how the translational mechanisms of TELMS play a role in individual-level learning. Although the intervention used a traditional, individual-level model of CE, we were able to identify the influence of mechanisms of change underlying TELMS principles, such as attitudes and beliefs about the application of evidence-aligned MS care, and the commitment and confidence to engage in multidisciplinary strategies for coordination of care and systems-based changes. Textual interpretation of data from interviews revealed how care providers act in various scenarios that parallel the learning stages of TELMS. These concepts were supported by qualitative approaches, which provided observations about the types of actions, engagement, and partnerships chosen by different types of healthcare providers working within complex delivery systems. Elements of the translational mechanisms proposed by TELMS were consistently observed in the narrative reflections, and practitioners drew substantially on these ideas when confronting challenges in delivery of MS care within diverse cultural and sociocultural settings. Advanced-practice clinicians and physician specialists had similar visions on approaches to improve awareness of deficits or gaps in evidence-based care delivery, enhance personal commitment among teams, convert information into practical actions, and engage patients, caregivers, and other healthcare providers.

Aligning treatment goals between the multidisciplinary care team and patients is essential for optimizing MS management and improving outcomes, although viewpoint differences on treatment goals can persist between patients and providers.^18^ Patients with MS frequently demand information to actively participate in shared decision making and self-management.^19^ Disparities between treatment goals put forth by healthcare providers and patients highlight the need for greater communication and cooperation at a systems level.^20^

Qualitative findings of this hypothesis-generating study provided insights that may be valuable for the development of future interventions. The inquiry revealed much about the learners’ perceptions and attitudes toward broader approaches to MS care, including coordination of care, multidisciplinary practice, systems-level change, and the economic impact of current practices. For example, barriers to implementation of systems change and care coordination were revealed by those seeking change in the organizational culture or trying to improve communication. When viewed collectively, the findings suggest that the learning activity stimulated inquiry and enthusiasm for enhanced collaboration and greater commitment to concrete behavioral changes to improve MS care.

One example of expanded systems-based considerations by learners was a notable interest in reducing costs and controlling the economic impact of MS through improved care. The learning activity on which this study was based included an interactive application to demonstrate the economic impact of MS relapse on costs, similar to those used in previous educational activities, which may have reinforced appreciation of the potential economic impact of improving care.^21^^,^^22^ After the activity, nearly half of healthcare providers reported that they had implemented systems-based care changes to reduce costs, a key element of the Triple Aim.

The results of this study should be viewed in the context of its limitations. Survey results for commitment to change are limited because they are self-reported. Nevertheless, self-reported commitment is a valid predictor of behavioral change in practice and provides a useful surrogate measure in the absence of observational data.^23^^,^^24^ Although the proportion of participants who responded to the post-activity 30-day survey was relatively small, it was representative of the activity completers in terms of profession and specialty and provided a useful indication of implementation relative to learning and stated commitment. The point of redundancy was reached in data collection, but saturation may have been limited, given the small sample size. One study evaluated the necessary sample size for a thematic analysis of a group of MS patients.^25^ The authors found that 12 interviews provided a sample size sufficient to achieve saturation with a heterogeneous sample. Similarly, another study of saturation and variability undertook 60 interviews and found saturation was observed after the first 12 interviews.^26^ The present study used a narrative method, and strived for illustrative or evocative sampling, rather than saturation.^27^ Accordingly, the results of the analysis are not intended to be generalizable or universal, but rather reflective of the participants. Future studies should be conducted to extend these findings to other settings providing MS care and to identify generalizable behavior change on a longitudinal basis to determine the long-term influence of education on clinical practice and patient outcomes.

In conclusion, elements of translational mechanisms underlying the process of continuous learning in TELMS were influential in a conventional educational intervention. Although the insights of TELMS emphasize the benefit of systems-based interventions that are integrated across the healthcare delivery system, our preliminary findings suggest that these principles are relevant to learners in provider-based learning activities. We found evidence that activity participants recognized the mechanisms of behavior change that are important in continuous learning. Further, the activity participants demonstrated commitment and practice changes related to improving awareness, converting information, pursuing engagement, and developing partnerships. Commitment to change and self-reported behavioral change demonstrated that learners enhanced their engagement in systems-levels practice change.

### Acknowledgements

We are grateful to the clinician learners who participated in the interviews. The educational intervention and qualitative study were supported in part by an educational grant from Genentech, Inc.

### Conflict of Interest

The authors declare that they have no conflict of interest.
